# Axon Guidance-Related Factor FLRT3 Regulates VEGF-Signaling and Endothelial Cell Function

**DOI:** 10.3389/fphys.2019.00224

**Published:** 2019-03-12

**Authors:** Suvi Jauhiainen, Johanna P. Laakkonen, Kirsi Ketola, Pyry I. Toivanen, Tiina Nieminen, Takeshi Ninchoji, Anna-Liisa Levonen, Minna U. Kaikkonen, Seppo Ylä-Herttuala

**Affiliations:** ^1^A.I. Virtanen Institute for Molecular Sciences, University of Eastern Finland, Kuopio, Finland; ^2^Rudbeck Laboratory, University of Uppsala, Uppsala, Sweden; ^3^Institute of Biomedicine, University of Eastern Finland, Kuopio, Finland; ^4^Heart Center and Gene Therapy Unit, Kuopio University Hospital, Kuopio, Finland

**Keywords:** angiogenesis, axon guidance factor, cell survival, endothelial cell, fibronectin-leucine-rich transmembrane protein, gene expression, gene expression regulation, vascular endothelial growth factor

## Abstract

Vascular endothelial growth factors (VEGFs) are key mediators of endothelial cell (EC) function in angiogenesis. Emerging knowledge also supports the involvement of axon guidance-related factors in the regulation of angiogenesis and vascular patterning. In the current study, we demonstrate that fibronectin and leucine-rich transmembrane protein-3 (FLRT3), an axon guidance-related factor connected to the regulation of neuronal cell outgrowth and morphogenesis but not to VEGF-signaling, was upregulated in ECs after VEGF binding to VEGFR2. We found that FLRT3 exhibited a transcriptionally paused phenotype in non-stimulated human umbilical vein ECs. After VEGF-stimulation its nascent RNA and mRNA-levels were rapidly upregulated suggesting that the regulation of FLRT3 expression is mainly occurring at the level of transcriptional elongation. Blockage of FLRT3 by siRNA decreased survival of ECs and their arrangement into capillary-like structures but enhanced cell migration and wound closure in wound healing assay. Bifunctional role of FLRT3 in repulsive vs. adhesive cell signaling has been already detected during embryogenesis and neuronal growth, and depends on its interactions either with UNC5B or another FLRT3 expressed by adjacent cells. In conclusion, our findings demonstrate that besides regulating neuronal cell outgrowth and morphogenesis, FLRT3 has a novel role in ECs via regulating VEGF-stimulated EC-survival, migration, and tube formation. Thus, FLRT3 becomes a new member of the axon guidance-related factors which participate in the VEGF-signaling and regulation of the EC functions.

## Introduction

Angiogenesis is indispensable for development, growth and regeneration of all tissues by supplying nutrients and oxygen (Persson and Buschmann, 2011). Sprouting angiogenesis is initiated when endothelial cells (ECs) in the vessel wall, called tip cells, are polarized leading to their extension and migration toward an angiogenic stimulus. After filopodial protrusion, other ECs in the vessel wall, called stalk cells, proliferate and form a cord for the developing sprout of the vessel. Elongation of the sprout continues until a tip cell reaches another tip cell or a small vessel and forms a new connection (Risau, 1997; Ribatti and Crivellato, 2012). Lumen formation is there after facilitated by intracellular vacuolization or cell–cell repulsion, i.e., by local repulsion of cell surfaces of the neighboring ECs in the newly formed non-lumenized cord (Kamei et al., 2006; Strilić et al., 2010). Finally, vessel maturation is promoted by recruitment of pericytes as well as assembly and production of basement membrane (Stratman et al., 2009; Davis et al., 2015).

Vascular endothelial growth factors (VEGFs) are key mediators of EC function during angiogenesis (Ylä-Herttuala et al., 2017). The VEGF family consists of at least seven members, including VEGF-A-F and placental growth factor (PlGF). VEGFs are secreted dimeric glycoproteins that function via three tyrosine kinase receptors, VEGFR-1 (Flt1), VEGFR-2 (KDR), and VEGFR-3 (Flt4). Most of the angiogenic responses, including vascular EC survival and sprouting, formation of tip cells and vascular tubes, are mediated via VEGFR-2, whereas VEGFR-1 serves as a reservoir and a decoy receptor for VEGF-A in ECs. VEGFR-1 also participates in actin-cytoskeleton organization and EC migration (Koch et al., 2011; Tugues et al., 2011). In normal physiological conditions, expression of VEGFR-3 is mainly restricted to lymphatic vasculature where it mediates lymphangiogenic signaling of certain VEGFs (Sáinz-Jaspeado and Claesson-Welsh, 2018).

Increasing evidence also supports the participation of axon guidance-related factors in the regulation of angiogenesis and vascular patterning (Adams and Eichmann, 2010). Here we have investigated axon guidance-related factors in ECs after stimulation with VEGFR-2 ligands and show for the first time that fibronectin and leucine-rich transmembrane protein-3 (FLRT3) is a novel target gene for VEGF-stimulated VEGFR-2 actions. FLRT3 is a transmembrane protein which was first identified in a screening of extracellular matrix components from human skeletal muscle cDNA libraries (Lacy et al., 1999). In addition to skeletal muscle, it is highly expressed in brain, kidney and lung, and with lower quantity in pancreas, heart, placenta and liver (Lacy et al., 1999). Prior to this study, FLRT3 has been shown to participate in repulsive and adhesive cellular guidance as well as fibroblast growth factor (FGF) signaling during embryogenesis and neuronal growth (Böttcher et al., 2004; Robinson et al., 2004; Chen et al., 2009; Karaulanov et al., 2009; Hampel et al., 2011). FLRT3 deletion is embryonic lethal in mice and leads to a variety of malformations, like disorganized basement membrane, failure of embryonic turning and ventral body closure, cardiac bifida and asymmetric development of headfolds (Egea et al., 2008; Maretto et al., 2008). Despite earlier studies, a role of FLRT3 in the regulation of EC function remains ill-defined and there is no previous data about its connection to VEGF-signaling.

## Results

### Axon Guidance-Related Factors Are Altered in Gene Chip Data

In Gene Chip data, we identified several axon guidance-related genes differentially expressed in AdVEGF-D^ΔNΔC^-transduced HUVECs as compared to control cells (Table 1). Besides important roles in neurite outgrowth, many of these genes have been connected to EC biology. Neuropilin (NRP) -2 functions as a co-receptor for VEGFs and it stabilizes or enhances the binding of VEGFs to VEGFR tyrosine kinases (Staton et al., 2007). Type 3 semaphorins interact with NRP1 and NRP2 on cell surface and play a role as competitive inhibitors for the binding of VEGFs to these co-receptors (Gaur et al., 2009). Slit2-3 and their receptors (roundabouts, ROBOs) participate in the regulation of EC integrity and migration (Legg et al., 2008; Nieminen et al., 2015). Eph receptor tyrosine kinases and their transmembrane ephrin ligands transduce forward as well as reverse signaling in a cell-cell-dependent fashion and participate in the regulation of arterial-venous differentiation (Kuijper et al., 2007). Netrin-4 has a bifunctional action on ECs and depending on circumstances it can either promote or inhibit angiogenesis (Lejmi et al., 2008; Lambert et al., 2012).

**Table 1:** Axon guidance-related genes altered significantly at mRNA level in AdVEGF-D^ΔNΔC^-transduced HUVECs as compared to AdCMV-tranduced control cells.

Entrez ID	Symbol	Description	36 h	72 h
23767	FLRT3	Fibronectin-leusine-rich transmembrane protein 3	3,9	–
23768	FLRT2	Fibronectin-leusine-rich transmembrane protein 2	1,7	–
219699	UNC5B	Netrin receptor UNC5B	1,4	–
59277	NTN4	Netrin-4	-1,4	–
8828	NRP2	Neuropilin-2	1,6	–
56920	SEMA3G	Semaphorin-3G	-2,0	–
6405	SEMA3F	Semaphorin-3F	1,3	–
54910	SEMA4C	Semaphorin-4C	1,3	–
8482	SEMA7A	Semaphorin-7A	1,3	–
55558	PLXNA3	Plexin-A3 Precursor	1,3	–
9353	SLIT2	Slit homolog 2 protein	–	-1,9
6586	SLIT3	Slit homolog 3 protein	1,3	–
6091	ROBO1	Roundabout homolog 1	–	-1,4
1943	EFNA2	Ephrin-A2	–	1,3
1945	EFNA4	Ephrin-A4	1,3	–
1948	EFNB2	Ephrin-B2	-1,6	-1,5
2047	EPHB1	Ephrin type-B receptor 1	1,6	1,4
2050	EPHB4	Ephrin type-B receptor 4	1,3	–


*For the analysis, total-RNA was extracted from Ad-transduced cells 36h and 72h post-transduction and hybridized to Human Genome U133 Plus 2.0 GeneChips (Affymetrix). Data analysis for three replicate samples in each study group was performed using improved software most suitable for a small number of replicates. Fold changes are presented as log2 values of the differences between AdVEGF-D and AdCMV-transduced cells. *p* < 0.05 was considered as a significant alteration in gene expression. -, no alteration in the mRNA expression level between AdVEGF-D-transduced and control cells*.

The most notable upregulated group of axon guidance factors in the Gene Chip data were FLRT2, FLRT3 and their receptor UNC5B (Table 1). UNC5B is known to be expressed in ECs, especially in tip cells of the sprouting vessels (Autiero et al., 2004). Interestingly, recent study by Seiradake et al. (2014) demonstrates that, in addition to their expression in neuronal cells, substantial level of FLRT2 and FLRT3 was also detected in vascular ECs. Yet, a linkage between FLRTs and VEGF-signaling has not been previously reported.

### Transcriptional Landscapes of FLRT2, FLRT3, and UNC5B Loci – FLRT3 Gene Displays a Transcriptionally Paused Phenotype

**FIGURE 1:**
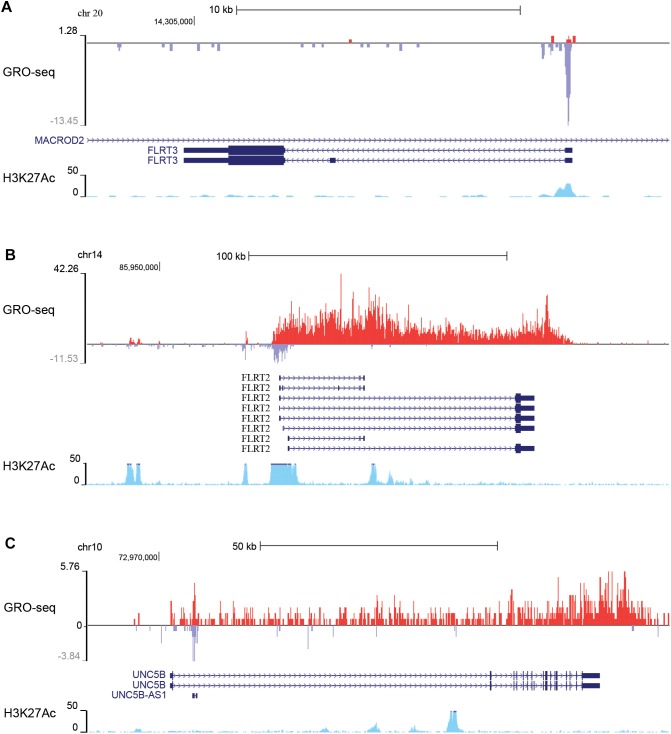
GRO-seq data from non-stimulated HUVECs showing basal transcription of nascent RNA (red color, from +strand; blue color, from –strand) in the genomic regions where FLRT3 gene **(A)**, FLRT2 gene **(B)**, and UNC5B gene **(C)** are located. Increased acetylation of histone 3 lysine 27 (H3K27Ac; light blue) further confirms the active genomic regions: paused RNA polymerase at the promoter region of FLRT3 **(A)**, antisense RNA at the promoter as well as potential enhancers in non-coding/intronic regions 55 and 12 kb upstream and 36 kb downstream of the FLRT2 promoter **(B)**, and antisense RNA (UNC5B AS1) in a region close to UNC5B promoter as well as several intronic enhancers downstream from UNC5B promoter **(C)**.

To gain more insight into the expression and regulation of these genes, we analyzed global run on sequencing (GRO-seq) data collected from non-stimulated HUVECs (Kaikkonen et al., 2014) that measures the nascent transcription profiles of all RNAs genome-wide. FLRT3 locates on chromosome 20 and is transcribed from the negative -strand in a region overlapping with MACROD2 (Figure 1A), a gene which polymorphism has been associated with the risk of coronary artery disease and hypertension (Slavin et al., 2011). According to GRO-seq data, expression level of FLRT3 gene in non-stimulated HUVECs is very low (Figure 1A). Despite this fact, the presence of active histone mark acetylation of histone H3 lysine 27 (H3K27Ac) as well as a high peak of nascent RNA produced in the promoter region of FLRT3 (Figure 1A), indicated that FLRT3 is actively transcribed but exhibits a transcriptionally paused phenotype.

FLRT2 and UNC5B locate in chromosomes 14 and 10, respectively. In HUVECs, they are transcribed from the +strand in relatively high quantities even without stimulus and display no evident promoter proximal pausing (Figure 1B,C). FLRT2 gene further displays production of an antisense RNA originating from the promoter region as well as expression of enhancer RNAs (i.e., bidirectional transcription of nascent non-coding RNA and enriched H3K27Ac) in regions 55 and 12 kb upstream and 36 kb downstream of the promoter (Figure 1B). Likewise, in UNC5B gene there is a transcription of the promoter associated antisense RNA (UNC5B AS1) as well as several intronic enhancers downstream from the UNC5B promoter (Figure 1C). These non-coding RNAs have potential ability to regulate the expression of FLRT2 and UNC5B.

### FLRT3 Is Upregulated in HUVECs via a VEGFR-2-Dependent Mechanism

**FIGURE 2:**
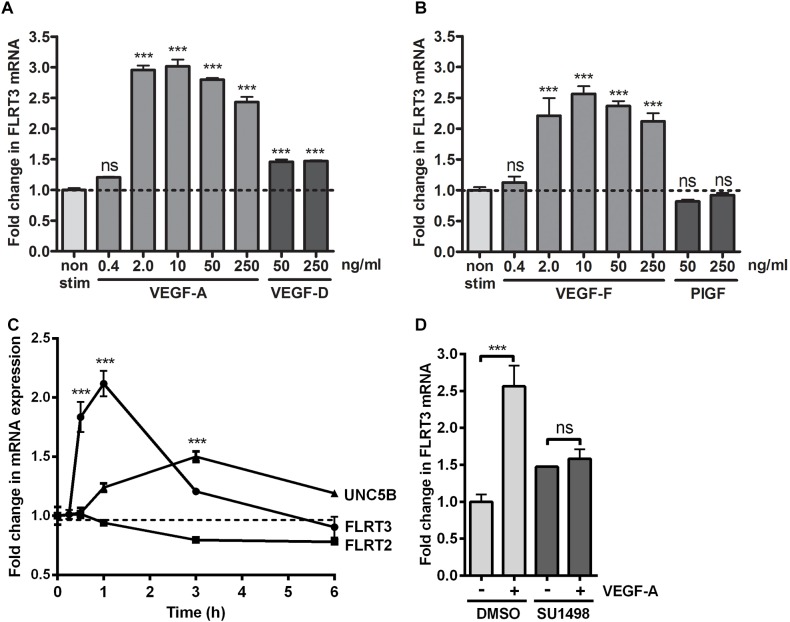
FLRT3 was upregulated in HUVECs via vascular endothelial growth factor (VEGFR)-2-dependent pathway. **(A,B)** Stimulation of HUVECs with different doses of VEGFR-2-binding ligands VEGF-A, VEGF-D^ΔNΔC^, VEGF-F and PlGF. qPCR measurement for FLRT3 mRNA was performed with target-specific assay-on-demand. **(C)** Upregulation of FLRT3 mRNA was a rapid response to VEGF-A (50 ng/ml) stimulation. A significant upregulation of UNC5B mRNA was also seen in VEGF-A-stimulated HUVECs; however, the response was weaker and had a slower kinetics. Expression of FLRT2 was not altered under these conditions. **(D)** SU1498, an inhibitor of VEGFR-2, abolished the VEGF-A-stimulated induction of FLRT3 mRNA in compared to DMSO-treated control cells. For all experiments, results are presented as mean ± SEM and are representative of 2–3 independent experiments done in triplicates. ^∗∗∗^*p* < 0.001; and ns, non-significant.

**FIGURE 3:**
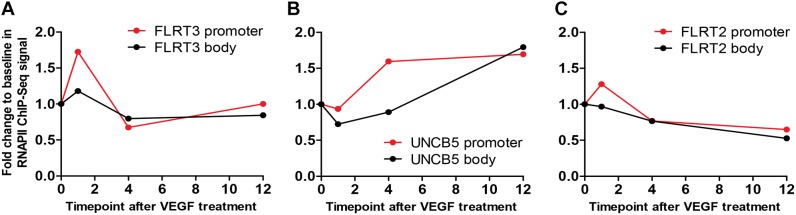
A time course analysis of RNA polymerase II (RNAPII) ChIP-Seq. **(A)** In FLRT3, a clear induction of signal at the promoter (red) and at the body (black) of the gene was seen 1h after VEGF-A-stimulation. **(B)** In-line with qPCR, VEGF-A-stimulated response to UNC5B gene was significant but slower and only evident 4 h post-treatment. **(C)** The signal at the promoter (red) and the body (black) of FLRT2 gene was not altered at any time point.

To confirm the effects of VEGFs on the expression of FLRT3, FLRT2 and UNC5B, HUVECs were stimulated with different rhVEGFs. In-line with GRO-seq data, basal expression level of the FLRT3 mRNA in HUVECs was very low. After stimulation, a significant upregulation of the FLRT3 mRNA was detected with VEGFR-2-binding ligands VEGF-A and VEGF-F at the concentrations 2–250 ng/ml and VEGF-D^ΔNΔC^ at 50 and 250 ng/ml (Figure 2A,B). VEGFR-1-binding ligand PlGF did not alter the expression of FLRT3 (Figure 2B). Induction of the FLRT3 mRNA started already 30 min after the stimulation with VEGF-A (50 ng/ml) and the highest upregulation of FLRT3 was seen 1–1.5 h post-treatment (Figure 2C). The results were further confirmed from a time course analysis of RNA polymerase II (RNAPII) ChIP-Seq, where a clear induction of signal at the promoter and at the body of the gene was seen 1h after VEGF-A-stimulation (Figure 3A). The rapid induction of gene expression is thus likely explained by a fast release of the paused polymerase into a productive elongation (Kaikkonen et al., 2014). Presence of SU1498, an inhibitor of VEGFR-2, in cell culture medium was able to abolish the induction of FLRT3 mRNA by VEGF-A-stimulation in comparison to DMSO controls (Figure 2D) which confirms the importance of VEGFR-2 in this process. VEGF-A-induced upregulation of UNC5B was also significant; however, the response was slower and only evident 3–4 h after the VEGF-A treatment (Figure 2C, 3B). No alteration in FLRT2 mRNA was observed under these conditions (Figure 2C, 3C).

**FIGURE 4:**
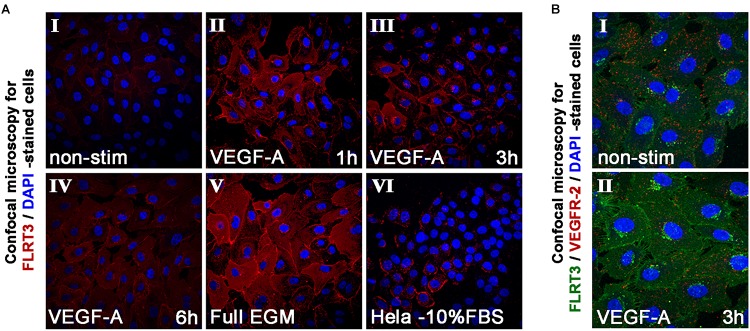
Immunofluorescent staining of FLRT3. **(A)** Immunofluorescent staining of FLRT3 confirmed VEGF-A-induced upregulation of FLRT3 also at the protein level and its internalization and localization from cell surface into cytoplasm and small intracellular vesicles near the nucleus (panels I–IV, representative pictures of non-stimulated and VEGF-A-stimulated HUVECs at 1–6 h time points). A part of the positivity for FLRT3 was retained also at cell surface, especially on areas where adjacent HUVECs were in contact to each other (panels II–III). Higher expression of FLRT3 was detected in proliferating HUVECs grown in high serum conditions (V). Hela cells expressing low quantity of endogenous FLRT3 were used as negative controls for the immunofluorescent stainings (VI). **(B)** Immunofluorescent double-staining for FLRT3 and VEGFR-2 in non-stimulated and VEGF-A-stimulated HUVECs 3 h post-treatment.

In addition to mRNA measurements, the expression of FLRT3 protein was detected by immunofluorescence staining and confocal microscopy. Non-stimulated HUVECs grown in low-serum conditions expressed a very low level of FLRT3 protein, which mostly localized in the cell surface (Figure 4A, panel I). More intense staining of FLRT3 was seen in the VEGF-A-stimulated HUVECs 1–6 h post-treatment (Figure 4A, panels II–IV) as well as in the proliferating HUVECs cultured in high-serum conditions (Figure 4A, panel V). At 1 h, a diffuse expression of FLRT3 was seen mainly on the cell surface and in the cytoplasm (Figure 4A, panel II). However, after 3 h post-treatment the FLRT3 expression was seen to localize in small intracellular vesicles near the nucleus (Figure 4A, panel III). HeLa cells grown in high-serum conditions were used as controls (Egea et al., 2008) and showed only a low level expression of FLRT3 protein on the cell surface (Figure 4A, panel VI).

### UNC5B and FLRT3 Are the Most Potent Binding Partners for FLRT3 in HUVECs

Intracellular trafficking of VEGFR-2 into the vesicles near the nucleus has been seen in ECs in response to VEGF-A-stimulation (Lampugnani et al., 2006). To test whether FLRT3 could co-localize in the same vesicles and have a functional interaction with the VEGFR-2, HUVECs were stimulated with VEGF-A (50 ng/ml). At 1 h, a decreased presence of VEGFR-2 on the cell surface was seen which is in line with the earlier findings (Lampugnani et al., 2006). At 3 h, double-staining with antibodies against FLRT3 and VEGFR-2 showed internalization of both proteins from plasma membrane into the cytoplasm. However, their co-localization was not detected at any of the tested time points (Figure 4B, panels I–II). This suggests that even though the activation of VEGFR-2 causes a rapid increase in FLRT3 expression, these two factors locate in separate cellular compartments.

To better elucidate the binding partners of FLRT3 in HUVECs, we further exploited the Gene Chip data from VEGF-transduced HUVECs. According to literature, potential binding partners for FLRT3 include UNC5B, FGF-receptor (FGFR) -1 and -2, ROBO-1 and latrophilins. Homogenic FLRT3-FLRT3 interactions between two FLRT3 molecules expressed by adjacent cells have also been suggested (Böttcher et al., 2004; Haines et al., 2006; Karaulanov et al., 2009; Leyva-Díaz et al., 2014; Seiradake et al., 2014; Jackson et al., 2015). Among these, ROBO-1 (Table 1) and latrophilin-1 (LPHN1; data not shown) were downregulated in the Gene Chip data at 72 h. FGF or FGFR levels were not altered in the Gene Chip data; however, transcriptional target genes for FGFR-1 signaling were downregulated at 72 h (data not shown).

**FIGURE 5:**
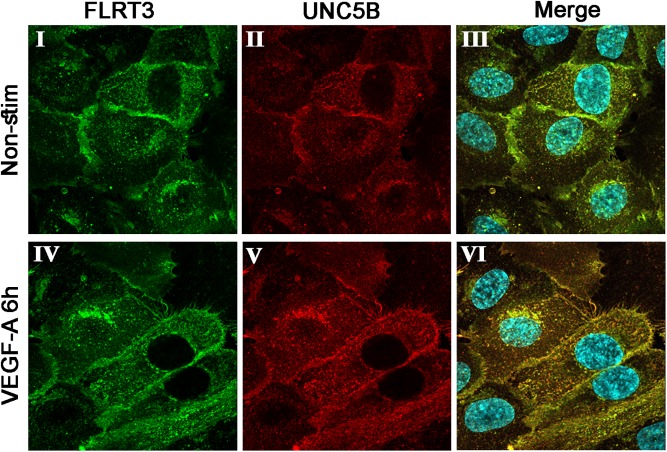
Immunofluorescent double-staining for FLRT3 and UNC5B in non-stimulated (I–III) and VEGF-A-stimulated (IV–VI) HUVECs 6 h post-treatment. Co-localization of these factors was detected especially in VEGF-A-stimulated HUVECs and took place on cell surface as well as intracellular vesicles near the nucleus (VI). Immunofluorescent stainings for FLRT3 (I, IV), UNC5B (II, V), and merged images including nuclear staining (blue color; III, VI).

As already listed, UNC5B was upregulated in Gene Chip data at 36 h (Table 1) and in the qPCR data after stimulation of HUVECs with VEGF-A protein (Figure 2C, 3B). To confirm the interactions between UNC5B and FLRT3 also in HUVECs, we performed double-staining for non-stimulated and VEGF-A-stimulated (50 ng/ml) HUVECs with specific antibodies. Immunofluorescent staining demonstrated that FLRT3 and UNC5B has significant co-localization in HUVECs, especially after VEGF-A-stimulation (Figure 5). Co-localization takes place on cell surface as well as in the intracellular vesicles near the nucleus (Figure 5, panel VI). Off note, upregulation of UNC5B protein was also evident in VEGF-A-stimulated cells 6 h post-treatment (Figure 5), confirming the findings from Gene Chip data and qPCR. Furthermore, in confocal microscopy, more intense cell-surface staining for FLRT3 was detected in cell–cell contact sites as compared to the cell-surface areas not in contact to other cells (Figure 4A, panels II–III; Figure 5). This suggests that FLRT3 molecules expressed by adjacent HUVECs after VEGF-A-stimulus likely interact with each other. Thus, the most prominent binding partners for FLRT3 in HUVECs are UNC5B and another FLRT3 molecule expressed by adjacent cells.

### VEGF-A-Induced Upregulation of FLRT3 Is Evident Also in Microvascular ECs

**FIGURE 6:**
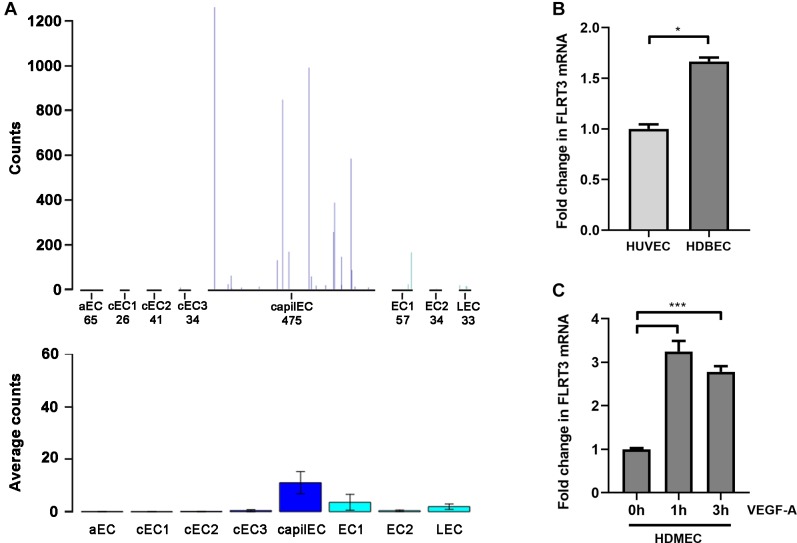
VEGF-A-stimulated upregulation of FLRT3 was detected also in microvascular ECs. **(A)** Single-cell transcriptomic data from mouse lung tissue show notably higher expression level of FLRT3 mRNA in capillary ECs than other EC clusters. **(B)** Comparative qPCR analysis for basal expression level of FLRT3 mRNA in HUVECs and microvascular ECs (HDMEC). **(C)** Significant upregulation of FLRT3 mRNA was detected in HDMECs 1 and 3 h after VEGF-A-stimulation. **(B,C)** The data presented as mean ± SEM are representative of repeated independent experiments done in triplicates. ^∗^*p* < 0.05; ^∗∗∗^*p* < 0.001.

Although expression of tip and stalk cell markers can be detected in VEGF-stimulated macrovascular ECs, like in HUVECs (Harrington et al., 2008), microvascular/capillary ECs are major players in developmental sprouting angiogenesis and tumor angiogenesis *in vivo*. Importantly, Vascular Single Cell database (He et al., 2018; Vanlandewijck et al., 2018) shows higher expression level of FLRT3 mRNA in mouse lung capillary ECs than other lung-derived EC clusters (Figure 6A). In our qPCR measurements, however, only 1.7-fold higher expression of FLRT3 mRNA was detected in human dermal microvascular ECs (HDMECs) than in HUVECs in low-serum conditions without external stimulus (Figure 6B). More importantly, a similar pattern for VEGF-A-induced upregulation of FLRT3 mRNA was detected in HDMECs (Figure 6C) as in HUVECs (Figure 2A,C).

### Role of FLRT3 in ECs *in vivo*

**FIGURE 7:**
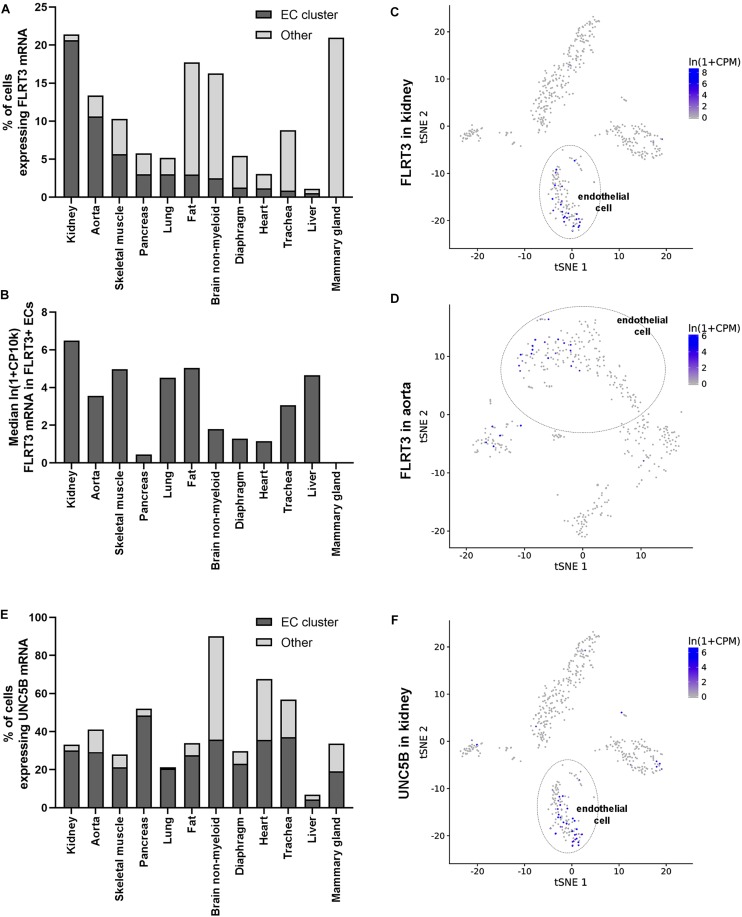
Expression of FLRT3 in ECs *in vivo*. **(A,B)** Single-cell sequencing data shows that FLRT3 mRNA is expressed in EC cluster of most of the mouse tissues included in the database (Tabula Muris Consortium et al., 2018). In addition, cells positive for FLRT3 mRNA were detected in several other cell clusters in all the tissues. **(A)** Percentage of ECs (dark gray) and other cells (light gray) positive for FLRT3 mRNA in each tissue. **(B)** Relative expression level of FLRT3 mRNA in FLRT3-positive ECs. **(C,D)** t-SNE plots showing the expression of FLRT3 mRNA based on PCA clustering of kidney **(C)** and aorta **(D)**. **(E)** Percentage of ECs positive for UNC5B mRNA in mouse tissues. **(F)** t-SNE plots showing the relative distribution of UNC5B mRNA in the kidney.

To gain more insight into the expression of FLRT3 in ECs *in vivo*, we took advantage on single-cell sequencing data by Tabula Muris Consortium (Tabula Muris Consortium et al., 2018). It was found that FLRT3 is expressed in EC cluster of most of the analyzed mouse tissues (Figure 7A–D). A portion of ECs positive for FLRT3 mRNA varied from 0.6 to 20.6% in different tissues (Figure 7A); kidney (Figure 7C) and aorta (Figure 7D) showing the highest quantity of FLRT3-positive ECs. Median relative expression level of FLRT3 mRNA in FLRT3-positive ECs varied from 0.45 to 6.5 (Figure 7B). Noteworthy, positivity for FLRT3 mRNA was not specific for ECs but several other cell clusters in each tissue expressed FLRT3, as well (Figure 7A,C,D). In addition, a clear positivity for UNC5B mRNA, the receptor for FLRT3, was detected in EC cluster in a variety of mouse tissues (Figure 7E,F). In-line with GRO-seq data and qPCR measurements done in HUVECs, higher positivity for UNC5B than for FLRT3 was detected in mouse tissues in these normal physiological conditions (Figure 7A,E), supporting the idea for a low basal expression of FLRT3.

### Inhibition of FLRT3 by siRNA Decreases EC Survival and Tube Formation *in vitro*

**FIGURE 8:**
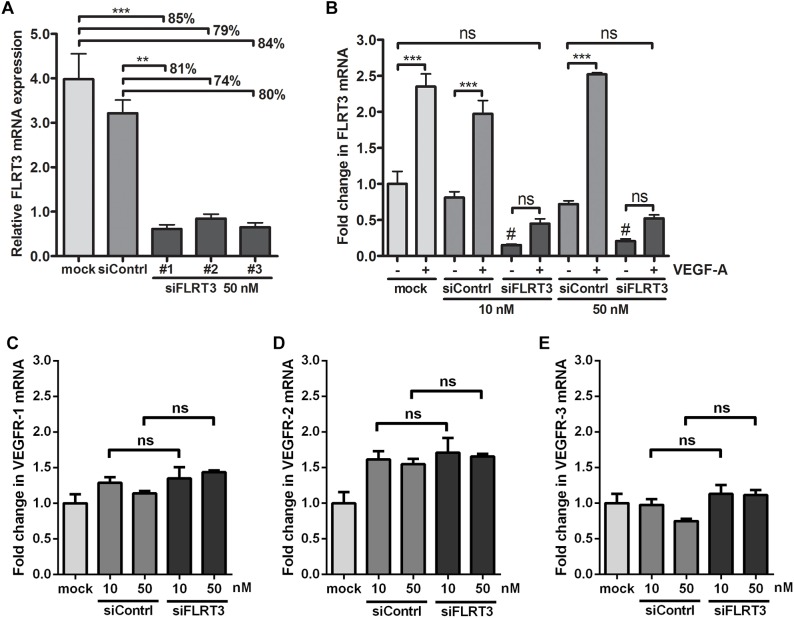
Efficiency of siRNA oligonucleotides targeting to FLRT3. **(A)** Three specific siRNA oligonucleotides targeting FLRT3 were tested. **(B)** FLRT3 siRNA #1 was chosen for further studies and it was able to cause a strong inhibition in the expression of FLRT3 mRNA in non-stimulated HUVECs as well as in the cells stimulated with VEGF-A (50 ng/ml) at both of the tested concentrations but did not affect VEGFR-1 **(C)**, VEGFR-2 **(D)**, or VEGFR-3 **(E)** expression in non-stimulated cells. The data presented as mean ± SEM are representative of repeated independent experiments done in 3–5 replicates. ^∗^*p* < 0.05; ^∗∗^*p* < 0.01; ^∗∗∗^*p* < 0.001; #*p* < 0.01 as compared to corresponding cells transfected with mock or control siRNA; and ns, non-significant.

To further study the function of FLRT3 in ECs, blocking experiments with siRNA oligonucleotides were performed. Three specific siRNA oligonucleotide sequences targeting FLRT3 were tested for mRNA silencing. All of them showed a significantly reduced expression of FLRT3 mRNA in non-stimulated conditions 48 h post-transfection (79–85 or 74–81% reduction in FLRT3 mRNA as compared to that of mock or control siRNA -transfected cells, respectively) (Figure 8A). FLRT3 siRNA #1 was chosen for further experiments, as it significantly inhibited FLRT3 mRNA production under basal conditions as well as after VEGF-A-stimulation at the tested concentrations (10 and 50 nM) (Figure 8B). Furthermore, FLRT3 siRNA #1 did not affect VEGF receptor mRNA expression (VEGFR-1-3) in non-stimulated conditions as compared to mock or control siRNA-transfected cells (Figure 8C–E) nor did it cause an interferon response (data not shown).

**FIGURE 9:**
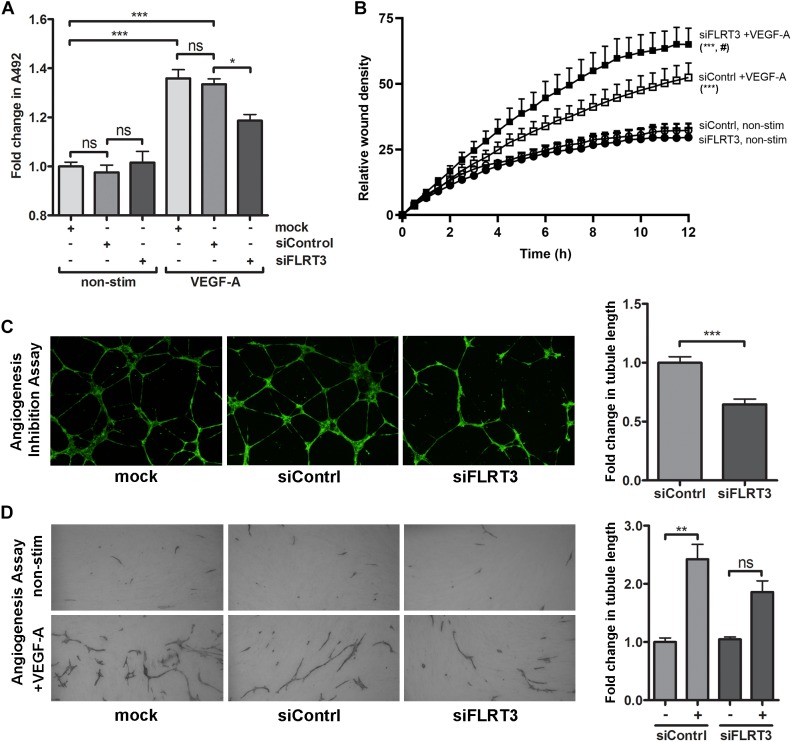
Transcriptional inhibition of FLRT3 in HUVECs decreased VEGF-A-stimulated cell survival and *in vitro* angiogenesis but enhanced cell migration. **(A)** In MTS assay, the ability of VEGF-A (50 ng/ml) to stimulate EC survival was significantly decreased in the HUVECs transfected with siRNA against FLRT3. **(B)** VEGF-A-stimulated cell migration, assessed as a relative wound density (RWD), was significantly enhanced over the time in HUVECs transfected with FLRT3 siRNA as compared to control siRNA-treated control cell. Non-stimulated HUVECs for both study groups was included to see the background level of RWD in each time point and cellular migration was monitored continuously using the IncuCyte S3 Live-cell Imaging System. **(C,D)** FLRT3 siRNA significantly decreased tube formation in *in vitro* angiogenesis models. The data presented as mean ± SEM are representative of repeated independent experiments done in 3–8 replicates. For the experiments in **(A,C,D)** ^∗^*p* < 0.05; ^∗∗^*p* < 0.01; ^∗∗∗^*p* < 0.001; #*p* < 0.01 as compared to corresponding cells transfected with control siRNA; and ns, non-significant. **(B)** Statistical significance between the study groups was quantified by comparing the main column effects over the time. ^∗∗∗^, #*p* < 0.001 as compared to corresponding non-treated cells or to the VEGF-A-stimulated cells transfected with control siRNA, respectively.

For the blocking experiments, 10 nM concentrations of FLRT3 siRNA and control siRNA were used. In MTS-assay, a significant increase in cell survival was observed in the wells stimulated with VEGF-A (50 ng/ml). VEGF-A-stimulation clearly induced EC survival also in cells transfected with FLRT3 siRNA; however, the response was significantly (*p* < 0.05) lower than in mock or control siRNA-transfected HUVECs (Figure 9A). Similar pattern was achieved in angiogenesis inhibition assay and EC tube formation assay where a decreased ability of ECs to arrange into capillary-like structures was seen in the wells transfected with FLRT3 siRNA (as compared to mock and control siRNA-transfected cells; Figure 9C,D). This implies that FLRT3 has a significant functional role in the regulation of EC survival and *in vitro* tube formation.

On contrary to these findings, in wound healing assay, transcriptional inhibition of FLRT3 significantly enhanced VEGF-A-stimulated migration of HUVECs as compared to control siRNA-transfected HUVECs (Figure 9B). Comparison between the study groups was done over 12-h period by using IncuCyte Life-Cell Imaging System. No difference between FLRT3 and control siRNA-transfected cells was detected in non-stimulated conditions (Figure 9B). A similar pattern for increased cell migration was achieved with transcriptional inhibition of UNC5B (data not shown). This gives added value for the involvement of UNC5B in the FLRT3-mediated EC functions.

## Discussion

FLRT3 is a transmembrane protein belonging to axon guidance-related factors. Prior to this study, it has been shown to regulate neuronal cell outgrowth after injury of peripheral nerves as well as to mediate embryonic cell adhesion and FGF-signaling during embryogenesis (Böttcher et al., 2004; Robinson et al., 2004; Chen et al., 2009; Karaulanov et al., 2009; Hampel et al., 2011). Here, we show for the first time that FLRT3: (1) exhibits a paused phenotype in non-stimulated HUVECs, (2) is rapidly upregulated in ECs after stimulation of VEGFR-2 with VEGFs; and (3) has a role in the regulation of VEGF-induced survival and migration of ECs as well as *in vitro* angiogenesis.

Vascular and nervous systems are both highly organized networks which especially in peripheral tissues usually develop in a coordinated fashion as two parallel systems (Adams and Eichmann, 2010). This is beneficial for both systems, as neuronal cells are dependent on oxygen and nutrients from the circulation, and in turn, arteries and arterioles are subjected to the regulation of vascular tonus by sympathetic nerves. Furthermore, formation of filopodial protrusions in a tip cell of sprouting capillaries as well as reorganization of actin cytoskeleton resembles the mechanism of axonal cone growth. Thus, it is not surprising that many molecules involved in axonal guidance and neurite outgrowth, e.g., NRPs, semaphorins, netrins, Slits, ROBOs, ephrins, and Eph receptors have been shown to be important in vascular patterning and sprouting angiogenesis (Kuijper et al., 2007; Staton et al., 2007; Legg et al., 2008; Lejmi et al., 2008; Gaur et al., 2009; Lambert et al., 2012; Nieminen et al., 2015). However, prior to this study, the role of FLRT3 in the regulation of EC function has not been well-defined and there is no previous data about its connection to VEGF-signaling. During embyogenesis and neuronal growth, FLRT3 mainly participates in repulsive as well as adhesive signaling (Böttcher et al., 2004; Robinson et al., 2004; Chen et al., 2009; Karaulanov et al., 2009; Hampel et al., 2011). Importantly, repulsive and adhesive signaling takes place also during angiogenesis and vascular remodeling by regulating tip cell elongation and migration (to define and fine-tune the direction and length of the neovascular sprout), lumen formation as well as prior re-establishment of EC-EC junctions. Increased knowledge about participation of FLRT3 to these EC functions is valuable.

Among VEGF-regulated genes in ECs, 40–60% portion has been shown to be transcriptionally paused, i.e., to have stoped RNA polymerases at their promoters (Gaertner and Zeitlinger, 2014; Kaikkonen et al., 2014). In these genes, the rate-limiting step for transcription is a recruitment of positive elongation factors (EFs) in the transcription complex or a release of negative EFs from the complex. Thus, after a positive stimulus, transition into the productive elongation can be initiated much quicker than in the genes where transcription initiation starts by the recruitment of RNA polymerase (Guo and Price, 2013; Kwak and Lis, 2013; Gaertner and Zeitlinger, 2014). Furthermore, the GRO-seq data from non-stimulated HUVECs demonstrated a high peak of nascent RNA transcription at the promoter of the FLRT3 gene. This is a feature commonly seen in the genes which exhibit the transcriptionally paused phenotype (Guo and Price, 2013; Kwak and Lis, 2013; Gaertner and Zeitlinger, 2014; Kaikkonen et al., 2014). In this study, we found that FLRT3 gene exhibits a paused phenotype and the gene expression is very rapidly upregulated in HUVECs after VEGF-stimulus. Similar pattern for upregulation of FLRT3 in response to VEGF-A-stimulus was detected in microvascular ECs. Altogether, this suggests that release of the paused polymerase into the productive elongation represents the most likely mechanism allowing for a rapid FLRT3 gene activation. In two other related genes, FLRT2 and UNC5B, this feature was lacking and no rapid responses were evident.

FLRT3 protein structure consists of an N-terminal domain with 10 leucine-rich repeats, a fibronectin-like domain, a single-pass transmembrane domain and a short cytoplasmic tail. Leucine-rich repeat domain is the most important for the repulsive as well as adhesive signaling of FLRTs. It facilitates the interaction of FLRT family members (FLRT1-3) with their multiple binding partners: netrin receptors UNC5A-D, fibroblast growth factor receptors (FGFRs), ROBO1, latrophilin as well as FLRT-FLRT interactions between adjacent cells (Böttcher et al., 2004; Haines et al., 2006; Hampel et al., 2011; Wei et al., 2011; Leyva-Díaz et al., 2014; Seiradake et al., 2014; Jackson et al., 2015). Among these, our data and current knowledge (Haines et al., 2006; Maretto et al., 2008; Hampel et al., 2011; Seiradake et al., 2014; Korsensky and Ron, 2016) support the fact that interactions with UNC5B as well as another FLRT3 expressed by adjacent cell are the most important ones particularly for FLRT3 in our model system. These heterogenic FLRT3-UNC5B and homogenic FLRT3-FLRT3 will be discussed in details below. A juxtamembrane linker region between fibronectin-like domain and transmembrane domain of FLRT3 contains a proteolytic cleavage site which promotes formation of a soluble FLRT3, capable of participating in the processes (Hampel et al., 2011; Seiradake et al., 2014).

UNC5B has a well-defined role in ECs and other cell types (Autiero et al., 2004; Larrivée et al., 2007; Lejmi et al., 2008; Karaulanov et al., 2009; Seiradake et al., 2014). Upregulation of UNC5B has been detected during sprouting angiogenesis and in the tip cells of growing capillaries (Autiero et al., 2004) and in our present study, after VEGF-stimulation. Ligand binding to UNC5B has been associated with a reduced adhesion of embryonic cells and repulsive neuronal and EC signaling, and in a case of Netrin-1 and -4, with anti-angiogenesis (Autiero et al., 2004; Larrivée et al., 2007; Lejmi et al., 2008; Karaulanov et al., 2009; Seiradake et al., 2014). In-line with the repulsive signaling, transcriptional inhibition of either FLRT3 or UNC5B was able to enhance VEGF-stimulated cell migration and wound closure in EC wound healing assay, i.e., to reduce repulsive signaling mediated by FLRT3-UNC5B interactions. Different functions for UNC5B have been proposed by Castets et al. (2009). They nominated UNC5B as a “dependence” receptor which leads ECs toward apoptotic signaling when no UNC5B-binding ligands are present (Castets et al., 2009). This has not been earlier connected to UNC5B-FLRT3 interactions; however, it could explain our findings from EC survival assay where transcriptional inhibition of FLRT3 by siRNA reduced VEGF-A-stimulated survival of ECs. Thus, data from us and others suggest that UNC5B is a likely receptor mediating the effects of FLRT3 not only in neuronal cells but in ECs as well.

**FIGURE 10:**
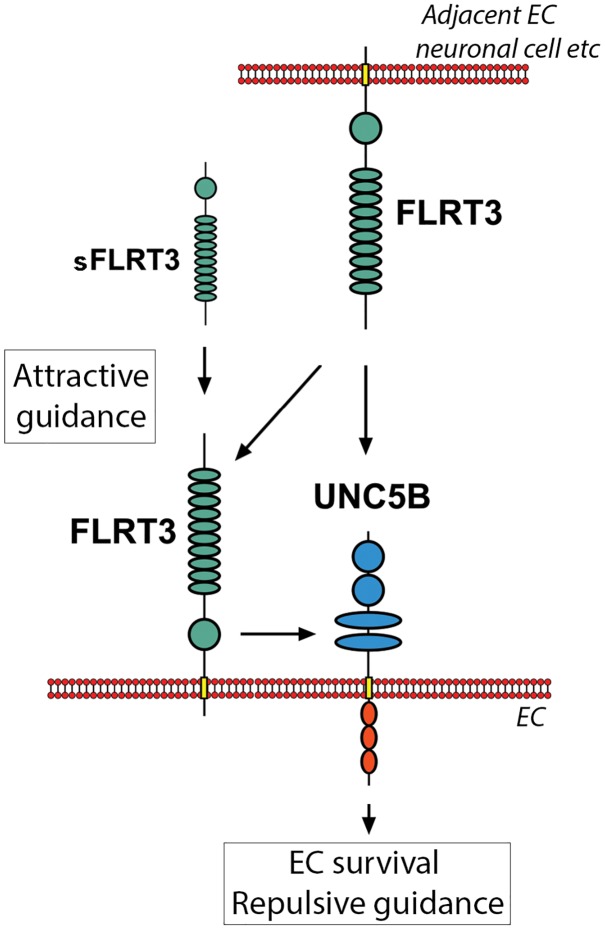
Scematic illustration showing interactions and downstream responses of FLRT3 with its key binding partners in the vasculature. Homogenic FLRT3-FLRT3 interactions (between FLRT3s expressed by adjacent cells or with soluble FLRT3) could promote attractive cellular guidance. Heterogenic FLRT3-UNC5B interactions, however, favor cell repulsion instead of attractive signaling. These competitive mechanisms may participate in fine-tuning vascular formation and angiogenesis. Furthermore, FLRT3 binding to UNC5B has a potential ability to switch off an apoptotic signaling promoted by UNC5B in the conditions deficient for UNC5B-binding ligands.

Homogenic FLRT3-FLRT3 interactions between adjacent cells have been shown to be important in cell-cell adhesion and attractive signaling during embryogenesis/neuronal growth (Robinson et al., 2004; Seiradake et al., 2014). As more intense cell-surface staining of FLRT3 was detected in VEGF-A-stimulated HUVECs in the areas where adjacent cells were in contact with each other, homogenic FLRT3-FLRT3 interactions likely takes place also in ECs and could participate in the EC functions. Interestingly, blockage of FLRT3 in ECs by siRNA-transfection significantly decreased tube formation in *in vitro* angiogenesis assays. In the case of siRNA-transfected HUVECs, we thus suggest a mechanism where lowered bioavailability of FLRT3 for FLRT-FLRT3 interactions would reduce adhesive signaling and consequently decrease elongation of EC tubules. Similarly to this, disturbance of FLRT3-FLRT3 interactions has been shown to decrease vessel sprouting in retinal explant cultures, while disturbance of FLRT3-UNC5B interactions had an opposing response (Seiradake et al., 2014). Seiradake et al. (2014) used modified soluble FLRT3 molecules in their model system, while we had transcriptional inhibition of FLRT3 directly in ECs. A schematic illustration for the homogenic FLRT3-FLRT3 interactions and heterogenic FLRT3-UNC5B interactions is shown in Figure 10.

Thus far, published data about expression and functions of FLRT3 in ECs *in vivo* are very limited. FLRT3 has been shown to be expressed in several highly vascularized tissues, i.e., in skeletal muscle, brain, kidney and lung, pancreas, heart, placenta, and liver (Lacy et al., 1999). Off note, expression of FLRT3 has prognostic value in certain cancers. In renal clear cell carcinoma, higher expression level of FLRT3 was associated with a better survival of the patients during a 5-years follow-up time (The Human Protein Atlas, 2018a), while in one of the less vascularized cancers, pancreatic cancer (Longo et al., 2016), a lower expression level of FLRT3 associates with better prognosis (The Human Protein Atlas, 2018b). Furthermore, by using non-direct method (b-galactosidase marker gene expressed under FLRT3 promoter in genetically modified mouse line), the expression of FLRT3 was suggested to take place in an inner plexiform layer of mouse retina, a tissue widely used to study angiogenesis (Seiradake et al., 2014). Here we took advantage of mouse single-cell sequencing databases (He et al., 2018; Tabula Muris Consortium et al., 2018; Vanlandewijck et al., 2018) and demonstrated that FLRT3 as well as UNC5B were both expressed in EC clusters of most of the mouse tissues included in the analysis, confirming its importance for EC function.

Taken together, VEGFs and their receptors are key mediators of EC functions, angiogenesis and lymphangiogenesis in physiological as well as in patophysiological conditions (Koch et al., 2011; Tugues et al., 2011; Ylä-Herttuala et al., 2017). Studies focusing on their target genes are required not only for the development of VEGF-based revascularization strategies for cardiovascular diseases but also for anti-VEGF therapies suitable for the treatment of cancer-related angiogenesis (Koch et al., 2011; Tugues et al., 2011; Ylä-Herttuala et al., 2017). Emerging knowledge supports the involvement of axon guidance-related factors in VEGF-signaling and vascular patterning (Kuijper et al., 2007; Staton et al., 2007; Legg et al., 2008; Lejmi et al., 2008; Gaur et al., 2009; Lambert et al., 2012). In this study we show that FLRT3 is a novel target gene for VEGF-stimulated VEGFR-2 actions with a role in the regulation of EC survival, migration and tube formation. Thus, FLRT3 becomes a new member of the axon-guidance factors which participates in the VEGF-signaling and the regulation of EC functions.

## Materials and Methods

### Materials

Serotype 5 adenovirus AdhVEGF-D^ΔNΔC^ contains hVEGF-D^ΔNΔC^ cDNA driven by a cytomegalovirus (CMV) promoter; AdCMV control virus contains the same CMV promoter and a poly(A) tail without a transgene. Recombinant human (rh) VEGF-A_165_ and rhPlGF were obtained from R&D Systems (Minneapolis, MN, United States). RhVEGF-D^ΔNΔC^ and rhVEGF-F were produced and purified as described previously (Jauhiainen et al., 2011; Nieminen et al., 2014). siRNA oligonucleotides targeting FLRT3 (#1: s24376, #2: s24377, and #3: s24378) and non-specific RNA control (Negative Control #1 siRNA) were obtained from Applied Biosystems (Life Technologies, Grand Island, NY, United States). Chemical inhibitor for VEGFR-2, Tryphostin SU1498, was provided from LC Laboratories (Woburn, MA, United States). HUVECs were isolated from umbilical cords with collagenase-treatment (Jauhiainen et al., 2011) and cultured up to passage 5 in Endothelial Cell Growth medium (Thermo Scientific, Rockford, IL, United States) on surface coated with 10 μg/ml fibronectin (Sigma-Aldrich, St. Louis, MO, United States) and 0.05% gelatin in phosphate buffered saline (PBS; Sigma-Aldrich). The Ethics Committee of the Kuopio University Hospital (Kuopio, Finland) has approved the collection of umbilical cords for cell isolation. Human dermal microvascular endothelial cells (PromoCell, Heidenberg, Germany) were cultured in Endothelial Cell Growth Medium MV2 (PromoCell) on surface coated with 10 μg/ml fibronectin and 0.05% gelatin. Hela cells (ATCC, Manassas, VA, United States) were cultured in Dulbecco’s Modified Eagles’ Media (DMEM; Gibco, Life Technologies) supplemented with 10% Fetal Bovine Serum (FBS; HyClone, Logan, UT, United States) and antibiotics.

### Gene Expression Arrays

Sample preparation and hybridization to Human Genome U133 Plus 2.0 GeneChips (Affymetrix, Santa Clara, CA, United States) has been described previously (Jauhiainen et al., 2011). Data analysis was performed using improved software most suitable for a small number of samples in each study group: data analyses were performed using R statistical software version 2.9.2 (R Foundation for Statistical Computing, Vienna, Austria) and Bioconductor version 2.4.1 (Bioconductor, Fred Hutchinsol Cancer Research Center, Seattle, WA, United States). Data was imported by Affy package version 1.11.8 (Bioconductor), using BrainArray CustomCDF version 12 custom Chip Description File (CDF) (The Psychiatry/MBNI MicroArray Lab, University of Michigan, Ann Arbor, MI, United States) for probe set matching and gene annotations. Quality assessment and control of the data was performed using Simpleaffy version 2.2 and AffyPLM version 1.2 packages (Bioconductor). Non-specific filtering was used to filter out less informative probe sets such as those not linked to genes or probe sets with a small variance across samples (50% of probe sets with the least variation) that are likely not expressed in the samples. Linear Models for Microarray Data (limma) version 2.18.3 analysis package (Bioconductor) was used to detect differentially expressed genes between the sample groups, using a linear fitting model(s) and empirical Bayes smoothing. Benjamini & Hochberg false discovery rate was used in analysis to adjust the data for multiple testing. Adjusted *p* < 0.05 was considered significant. For enrichment analysis of Gene Ontology terms and KEGG pathways, GOstats package version 2.1 (Bioconductor) was used.

### Stimulation of ECs With rhVEGFs

Confluent cultures of HUVECs or HDMECs kept in low-serum conditions [Endothelial basal medium (EBM, Thermo Scientific) supplemented with 0.5% FBS] for 16 h prior experimentation were stimulated with rhVEGF-A_165_, rhVEGF-D^ΔNΔC^, rhVEGF-F or rhPlGF at the concentrations 0.4–250 ng/ml for 0.5–6 h. For blocking experiments, 50 μM SU1498 or DMSO as a solvent control were added to the wells 30 min prior stimulation with VEGFs. Cells were harvested with Tri Reagent (Molecular Research Center, Inc., Cincinnati, OH, United States), total RNA was extracted and quantitative measurements of mRNA levels were performed using the Assays-on-Demand gene expression products (Table 2) (Applied Biosystems, Life Technologies) as described previously (Jauhiainen et al., 2011).

**Table 2:** Assay-on-demands used in qPCR measurement.

Gene	Description	Assay ID
B2M	Beta-2 microglobulin	Hs00187842_m1
FLRT3	Fibronectin-leucine-rich transmembrane protein 3	Hs00183798_m1
FLRT2	Fibronectin-leucine-rich transmembrane protein 2	Hs00544171_s1
UNC5B	Netrin receptor UNC5B	Hs00900710_m1
VEGFR-1	Vascular endothelial growth factor receptor-1	Hs01052961_m1
VEGFR-2	Vascular endothelial growth factor receptor-2	Hs00911700_m1
VEGFR-3	Vascular endothelial growth factor receptor-3	Hs01047677_m1


*The table includes abbreviation and description of target genes as well as Assay ID for the specific assay-on-demand gene expression product. Assay-on-demands were obtained from Applied Biosystems*.

### Confocal Microscopy

HUVECs grown on glass coverslips were fixed with 4% paraformaldehyde-PBS for 20 min at room temperature. Expression of FLRT3 was visualized using a goat polyclonal antibody against FLRT3 (1:50, R&D Systems) and a chicken anti-goat Alexa488 (Invitrogen, Life Technologies). VEGFR2 was detected with a rabbit monoclonal antibody for VEGFR2 (1:200, clone 55B11, Cell Signalling Technology, Beverly, MA, United States) and a goat anti-rabbit Alexa594 (Invitrogen, Life Technologies). UNC5B was detected with a rabbit polyclonal antibody against UNC5B (ab104871, 1:100, abcam, Cambridge, United Kingdom) and a goat anti-rabbit Alexa594 (Invitrogen, Life Technologies). Mounting was performed with ProLong Gold Antifade Reagent with DAPI (Life Technologies). LSM700 confocal microscope (Carl Zeiss, Jena, Germany) was used for imaging together with appropriate excitation and emission settings (488-nm argon laser and 543-nm HeNe-laser; 63x APO objective, NA 1.35; 512 × 512 pixels/image).

### Cell Survival and *in vitro* Angiogenesis Assays

MTS assay was performed as previously described (Jauhiainen et al., 2011). For Millicell μ-Angiogenesis Inhibition Assay (Millipore, Billerica, MA, United States) HUVECs transfected with siRNA targeting to FLRT3 or control siRNA were trypsinized 72 h post-transfection and seeded on ECMatrix Gel at 15,000 cells/well. After 16 h cells were stained with Calcein AM solution. Images of tubules were obtained with an Olympus IX71 microscope (Tokyo, Japan) using a 4x objective lens. Total tubule length in the microscopic fields taken from five replicate wells was quantified by using AnalySIS software (Soft Imagining System GmbH, Münster, Germany). V2A Kit (TCS Cellworks, Buckingham, United Kingdom) was performed according to manufacturer’s instructions. At day 2 cells were transfected with siRNA oligonucleotides. At day 4 fresh growth medium or growth medium supplemented with rhVEGF-A_165_ (10 ng/ml) was added into wells, repeating the procedure after each 2–3 days. After 14 days, cell cultures were fixed and stained for CD31 (according to manufacturer’s instructions). Tube formation was measured as the mean value from 10 to 12 microscopic fields from each cell culture well.

### Cell Migration Assay

Cell migration was assessed in a wound healing assay. HUVECs were seeded at 10,000 cells/well in a 96-well ImageLock plate (Essen BioSciences, Ltd., Hertfordshire, United Kingdom). Transfection with siRNA oligonucleotides was performed as previously described (Jauhiainen et al., 2011). 48 h post-transfection when cells reached confluency, low-serum medium (EBM supplemented with 0.5% FBS) was changed to the wells. After 16 h, HUVECs were stimulated with or without rhVEGF-A (50 ng/ml). A uniform scratch wound was generated in each well using the IncuCyte WoundMaker in ImageLock 96-well plates and the wound healing process was monitored continuously using the IncuCyte S3 Live-cell Imaging System (Essen BioSciences, Ltd., Hertfordshire, United Kingdom). Images were acquired every 30 min for a 24-h period using a 10X objective and analyzed using the IncuCyte Cell Migration Software module. Relative wound density (RWD) was used to quantify wound closure by comparing the mean RWD of 4–8 replicates. Two independent experiments were performed for the RWD studies and representative videos were generated using video generating tool of the IncuCyte Cell Migration Software module.

### Statistical Analysis

Results from repeated experiments are presented as means ± SEM. Statistical analysis was performed with GraphPad Prism 6.0 software (La Jolla, CA, United States) using Student’s *t*-test (Figure 6B, 9C), One-way ANOVA followed by Dunnett’s multiple comparison test (Figure 2A–D, 6C, 8A–E, 9A,D), or Two-way ANOVA followed by Tukey’s multiple comparison test (to compare main column effects between the study groups; Figure 9B). *p* < 0.05 was used to define a significant difference between the groups.

### NGS Experiments

Global run-on and library preparation for sequencing as well as GRO-seq data analysis has been described previously (Kaikkonen et al., 2014). The normalized data was visualized in UCSC genome browser. The public RNAPII ChIP-Seq was used to assess the changes in signal at the TSS and body of the gene by selecting the TSS and excluding the intragenic enhancers manually from the body of the genes. The hg19-coordinates used were: FLRT3 TSS (chr20: 14317956-14318395, strand -), FLRT3 body (chr20:14303414-14317866, strand -), FLRT2 TSS (chr14: 85994932-86000038, strand +), FLRT2 body (chr14: 86055063-86093801, strand +), UNC5B TSS (chr10:72971668-72973132, strand +), UNC5B body (chr10:73039507-73062283, strand +). The GRO-Seq data is available under GEO accession numbers GSE94872 and GSE52642. The RNAPII ChIP-Seq time course (0, 1, 4, 12 h) is available under accession GSE109625. Single cell RNA sequencing data from mouse tissues (He et al., 2018; Tabula Muris Consortium et al., 2018; Vanlandewijck et al., 2018) are available in http://betsholtzlab.org/VascularSingleCells/database.html, https://tabula-muris.ds.czbiohub.org/. Data was visualized by using user-friendly tools available in the above links. Histograms showing percentage of FLRT3 and UNC5B-positive cells in the FACS sorted cell clusters as well as median expression levels of FLRT3 in the positive cells were prepared by using GraphPad Prism 6.0 software.

## Data Availability

Publicly available datasets were analyzed in this study. These data can be found from the following links: https://www.ncbi.nlm.nih.gov/geo/query/acc.cgi?acc=GSE94872, https://www.ncbi.nlm.nih.gov/geo/query/acc.cgi?acc=GSE52642, http://betsholtzlab.org/VascularSingleCells/database.html, and https://tabula-muris.ds.czbiohub.org/.

## Author Contributions

SJ, JL, A-LL, MK, and SY-H planned the experiments. SJ performed cell culture experiments and qPCR measurements. SJ and PT processed and analyzed tube formation assay microscopy images. JL performed immunofluorescent staining and processed and analyzed confocal microscopy images. MK performed and analyzed GRO-seq experiments and analyzed RNAPII ChIP-Seq data. PT and TiN provided recombinant proteins. KK collected and analyzed data from the experiments done with IncuCyte. TaN helped to analyze the expression of FLRT3 *in vivo*. SJ, JL, A-LL, MK, and SY-H interpreted data and provided expert advice on methods. SJ, JL, KK, A-LL, MK, and SY-H participated in writing of the manuscript. All authors have red and approved the manuscript.

## Conflict of Interest Statement

The authors declare that the research was conducted in the absence of any commercial or financial relationships that could be construed as a potential conflict of interest.
